# The effect of adding lateral femoral cutaneous nerve block to pericapsular nerve group (PENG) block in hip surgery on postoperative morphine consumption: A randomized controlled trial

**DOI:** 10.1097/MD.0000000000044588

**Published:** 2025-09-19

**Authors:** Dinçer Firat Şeker, Keziban Bollucuoğlu, Çağdaş Baytar, Rahşan Dilek Okyay, Bengü Gülhan Köksal İncegül, Merve Sena Baytar, Özcan Pişkin, Hilal Ayoğlu

**Affiliations:** a Department of Anesthesiology and Reanimation, Zonguldak Bülent Ecevit University Medicine Faculty, Zonguldak, Turkey.

**Keywords:** lateral femoral cutaneous nerve block, opioid consumption, patient-controlled analgesia, pericapsular nerve group block

## Abstract

**Background:**

The lateral approach is commonly used in hip fracture surgery. Pericapsular nerve group (PENG) block cannot block the lateral femoral cutaneous nerve (LFCN), which is involved in sensing the skin incision during the lateral approach. Therefore, we compared the effect of adding the LFCN block to the PENG block on opioid consumption and pain scores in hip fracture operations under spinal anesthesia

**Methods:**

In this prospective randomized-controlled study, patients undergoing hip fracture surgery under spinal anesthesia were randomized into 3 groups: PENG, PENG + LFCN, and CONTROL group. In the PENG group, 20 mL of 0.25% bupivacaine was injected under ultrasound guidance, while in the PENG + LFCN group, LFCN block, involving 5 mL of 0.25% bupivacaine, was performed in addition to the PENG block. Spinal anesthesia was the preferred method in all patients. Postoperative opioid consumption, numerical rating scale (NRS) pain scores at 0-, 2-, 6-, 12-, and 24-hours postoperatively and while giving spinal anesthesia position, time of first analgesic requirement, and the time of first mobilization were recorded.

**Results:**

A total of 20 patients from each group were included in the statistical analysis. Postoperative opioid consumption was lower in the PENG and PENG + LFCN groups as compared to the CONTROL group, while the PENG and PENG + LFCN groups did not differ significantly (PENG: 8.10 ± 6.72 mg, PENG + LFCN: 8.40 ± 4.38 mg, CONTROL: 15.30 ± 5.59 mg, *P* < .001). Postoperative NRS pain scores during activity (NRS_A_) were significantly lower at all-time points in the PENG + LFCN than in the CONTROL group, and were lower at 2, 6, and 24 hours in the PENG than in the CONTROL group. These scores did not differ significantly between the PENG and PENG + LFCN groups at any time point. The time to first postoperative analgesic requirement was significantly shorter in the CONTROL than in the PENG and PENG + LFCN groups.

**Conclusion:**

Addition of an LFCN block to the PENG block did not contribute to postoperative opioid consumption and pain scores in patients undergoing hip fracture surgery under spinal anesthesia. Preoperative PENG block plays an important role both during positioning for spinal anesthesia and in postoperative analgesia management.

## 1. Introduction

Hip fractures, one of the leading causes of mortality and morbidity in older people, are common and usually require surgical repair, leading to severe pain that can result in complications, such as delirium, sleep disorders, and persistent pain during the perioperative period.^[1–4]^

Hip fracture pain increases with joint movement, and patients almost always experience discomfort when being positioned for neuraxial anesthesia techniques. This may reduce the chances of success of the technique by preventing the patient from assuming the optimal position. To alleviate this pain, paracetamol, nonsteroidal anti-inflammatory drugs, and opioids are commonly used.^[5,6]^ Opioids, particularly in older people, increase the risk of delirium, cause cognitive decline, and lead to side effects, including respiratory depression.^[7,8]^ Additionally, common concerns about the use of opioids include physical dependence and opioid-induced hyperalgesia. Opioid therapy aimed at pain relief can make patients more sensitive to pain and this has been associated with the dose of opioid used.^[9]^ Regional anesthesia and analgesia methods are alternative options that decrease opioid use and provide effective pain control.^[10]^

Pericapsular nerve group (PENG) block is a technique used for pain management in hip surgery and hip fractures, which can block all 3 nerves innervating the anterior part of the hip (femoral nerve, obturator nerve, and accessory obturator nerve) via a single injection, while minimizing the risk of motor block. However, since this technique does not affect the lateral femoral cutaneous nerve (LFCN), it has no effect on the pain caused by surgical incision.^[11,12]^

The research question of this study is: in patients undergoing hip fracture surgery under spinal anesthesia, does the addition of a LFCN block to a PENG block, compared with a PENG block alone or no block, reduce postoperative opioid consumption and improve pain outcomes? The primary outcome was postoperative opioid consumption, and the secondary outcomes included positioning and postoperative pain scores, time to first mobilization, length of hospital stay, and patient satisfaction.

## 2. Materials and methods

### 2.1. Study design

This prospective, randomized, controlled study was conducted between October 1, 2022, and January 31, 2024, in Zonguldak Bülent Ecevit University Hospital, Türkiye, after obtaining permission from the Local Ethics Committee (Protocol No: 2022/15-14, ClinicalTrials.gov identifier: NCT06226675). This study adhered to the CONSORT guidelines.

### 2.2. Study population

The study enrolled 60 patients between 18 and 75 years of age, classified as American Society of Anesthesiology Physical Status I to III, who underwent elective hip fracture surgery under spinal anesthesia.

Patients who refused to participate in the study, patients with local anesthetic allergy, coagulopathy, infection at the site of intervention, dementia/cognitive impairment, or American Society of Anesthesiology risk group intravenous (IV) and above were excluded. Written informed consent was acquired from patients. Patients were given a comprehensive explanation of the numerical rating scale (NRS) score for pain and the patient-controlled analgesia (PCA) device and its use. Patients were not involved in the design, conduct, or reporting of the study.

### 2.3. Randomization and blinding

The patients participating in the study were enrolled into 3 groups: CONTROL group, PENG group, and PENG + LFCN group, with the allocation determined by a computer-assisted program. The randomization process and the preparation of sealed envelopes were conducted by a staff member not involved in the study. All ward-based assessments and data collection were performed by another independent staff member who was blinded to the patients’ group allocation. Furthermore, the ward nurses were not provided with any information regarding the randomization. The anesthesiologist performing the blocks could not be blinded due to the nature of the intervention.

### 2.4. Block applications

Patients were taken to the block room prior to surgery where demographic data were recorded. Routine monitoring was performed. Pre-block NRS scores and hemodynamic parameters of the patients were evaluated and recorded. Patients not in the CONTROL group received 1 mg of midazolam for sedation before the block, whereas those in the CONTROL group received 1 mg of midazolam prior to spinal anesthesia. All blocks were performed by the same anesthesiologist (Dinçer Firat Şeker), who had prior experience of performing these blocks at least 50 times, under ultrasound guidance using a 22-gauge, 80-mm block needle.

#### 2.4.1. PENG block application

**Figure 1.**
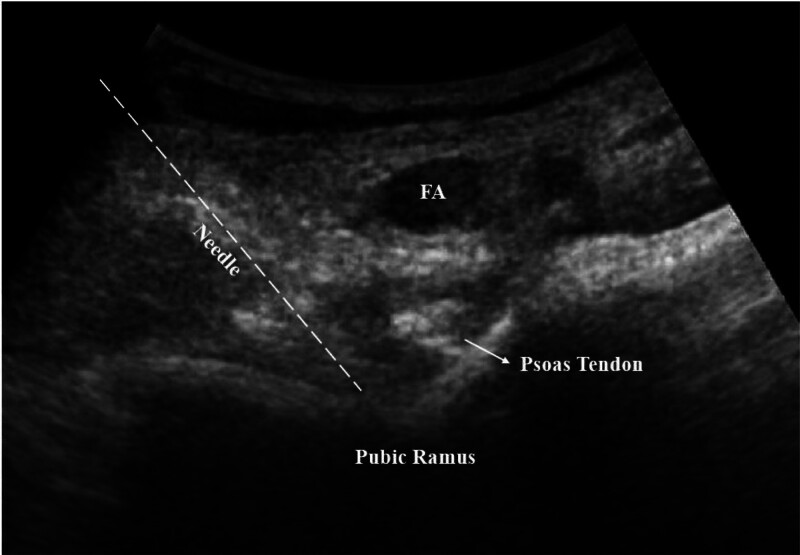
PENG block application. PENG = pericapsular nerve group.

Patients were placed in a supine position and the skin was cleaned using an antiseptic solution. The anterior inferior iliac spine was palpated and the convex probe was placed transversely onto the spine. Then, the probe was directed toward the symphysis pubis, making the iliopubic eminence, iliopsoas muscle and tendon, and femoral artery and vein visible. A 22-gauge, 80-mm block needle was inserted between the psoas tendon and the posterior pubic ramus, from the lateral to the medial direction, using the in-plane technique (Fig. 1). Following negative aspiration, 20 mL of 0.25% bupivacaine was injected and local anesthetic distribution was observed. The PENG blocks success was evaluated 20 minutes after the block using NRS scores. The NRS scores evaluated during 15-degree straight leg raising, hip flexion.

#### 2.4.2. LFCN block application

**Figure 2.**
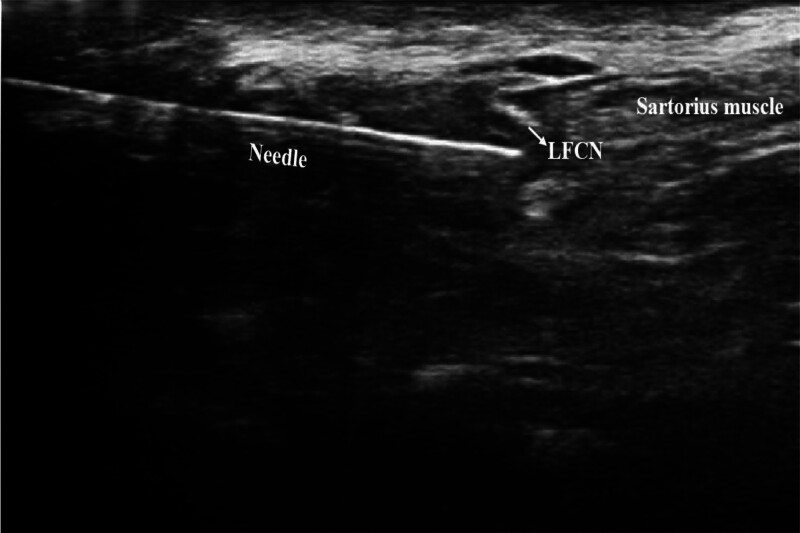
LFCN block application. LFCN = lateral femoral cutaneous nerve.

With the patient in the supine position, the skin was cleaned with antiseptic solution. A linear probe was placed parallel to the inguinal ligament. After identifying the femoral artery, vein and nerve, the sartorius and tensor fascia lata muscles were identified by advancing the probe laterally. Following identification of the nerve between the 2 muscles, a 22-gauge, 80-mm block needle was advanced from the lateral to the medial direction using the in-plane technique. When the needle tip was around the nerve, 5 mL of 0.25% bupivacaine was injected after negative aspiration and distribution of the local anesthetic around the nerve was observed (Fig. 2). Patients’ dermatomal coverage was evaluated 20 minutes after the block using a blunt-tipped needle. For successful lateral femoral cutaneous nerve block, a blunt-tipped needle was depressed on the proximal lateral thigh.

### 2.5. Anesthesia management

Spinal anesthesia was applied in all patients. Patients were placed in a sitting position and the NRS score was evaluated and recorded during positioning. Afterwards, the skin was cleaned with antiseptic solution and draped. A 25-gauge Quincke-tipped needle was inserted into the subarachnoid space from L3 to 4 or L4 to 5 with a median approach and the appropriate dose of 0.5% heavy bupivacaine was administered after free cerebrospinal fluid flow was observed. Patients were handed over to the surgical team after the block reached the T10 level. During the intraoperative period, routine hemodynamic parameters were measured and recorded at regular intervals. The anesthetist was unaware of the randomization.

### 2.6. Surgery procedure

All patients underwent minimally invasive intramedullary nailing in the supine position on the traction table after closed reduction by the same surgical team. Intraoperative complications (agitation, confusion, dizziness, dysphoria, dysarthria, tinnitus, auditory changes, metallic taste in the mouth, and seizure) were recorded. The duration of the operation and anesthesia were recorded at the end of surgery. Following the procedure, patients were transferred to the recovery unit.

### 2.7. Postoperative analgesic protocol

In the recovery room, an IV PCA device containing morphine was connected, which had a bolus dose of 2 mg without basal infusion, a lock-out time of 20 minutes, and a limit of 20 mg for 4 hours. Patients were transferred to the ward when the Modified Aldrete score was ≥9 in the recovery unit.

The resolution of motor block was defined as the the 0th hour. Patients received IV 1 g paracetamol every 8 hours and 20 mg of tenoxicam every 12-hour postoperatively. They were visited in their ward beds at 0-, 2-, 6-, 12-, and 24-h postoperatively to evaluate their pain at rest and during activity (15-degree straight leg raising, hip flexion). Pain was evaluated with the NRS score and recorded. Complications related to the block were recorded. The time to first mobilization and the time to first analgesic requirement were recorded. At the end of the 24th hour, IV PCA was terminated. The total amount of morphine consumed, as well as the number of bolus doses requested and administered, was recorded. Patient satisfaction was queried (1: very satisfied, 2: satisfied, 3: undecided, 4: not satisfied). The length of hospital stay after surgery (days) was recorded according to the discharge time.

### 2.8. Statistical analysis

The sample size was analyzed by using the G*Power programme with reference data from the study of Pascarella et al.^[13]^ According to the analysis based on our primary outcome, IV total morphine consumption, the minimum number of participants was calculated as 19 individuals per group in the sample size analysis performed with a 99% confidence interval and 95% power (P1: 4.0 [4.5], P2: 8.9 [4.0]). Considering potential case losses, 22 patients were included in each group.

Data analysis was performed using SPSS v20 software (SPSS IBM, Inc. Armonk). Descriptive variables are shown as mean ± standard deviation, median, minimum–maximum for quantitative data, and as frequency and % for qualitative data. The conformity of the quantitative values to a normal distribution in the groups was evaluated using the Shapiro–Wilk test. One-way analysis of variance was used to compare normally distributed continuous variables among the 3 groups. For variables showing a significant difference in the analysis of variance, multiple comparisons were conducted using the Tukey post hoc test. The Kruskal–Wallis test was applied for comparing non-normally distributed variables across the 3 groups. In cases where a significant difference was observed, pairwise comparisons were performed using the Dunn-Bonferroni post hoc test. To reduce the risk of type I error due to multiple comparisons, adjusted *P*-values were used in these tests. The Chi-square test was used for comparison of qualitative data between groups. The results of the analyses were evaluated with 95% confidence intervals. *P* < .05 was considered significant.

## 3. Results

**Figure 3.**
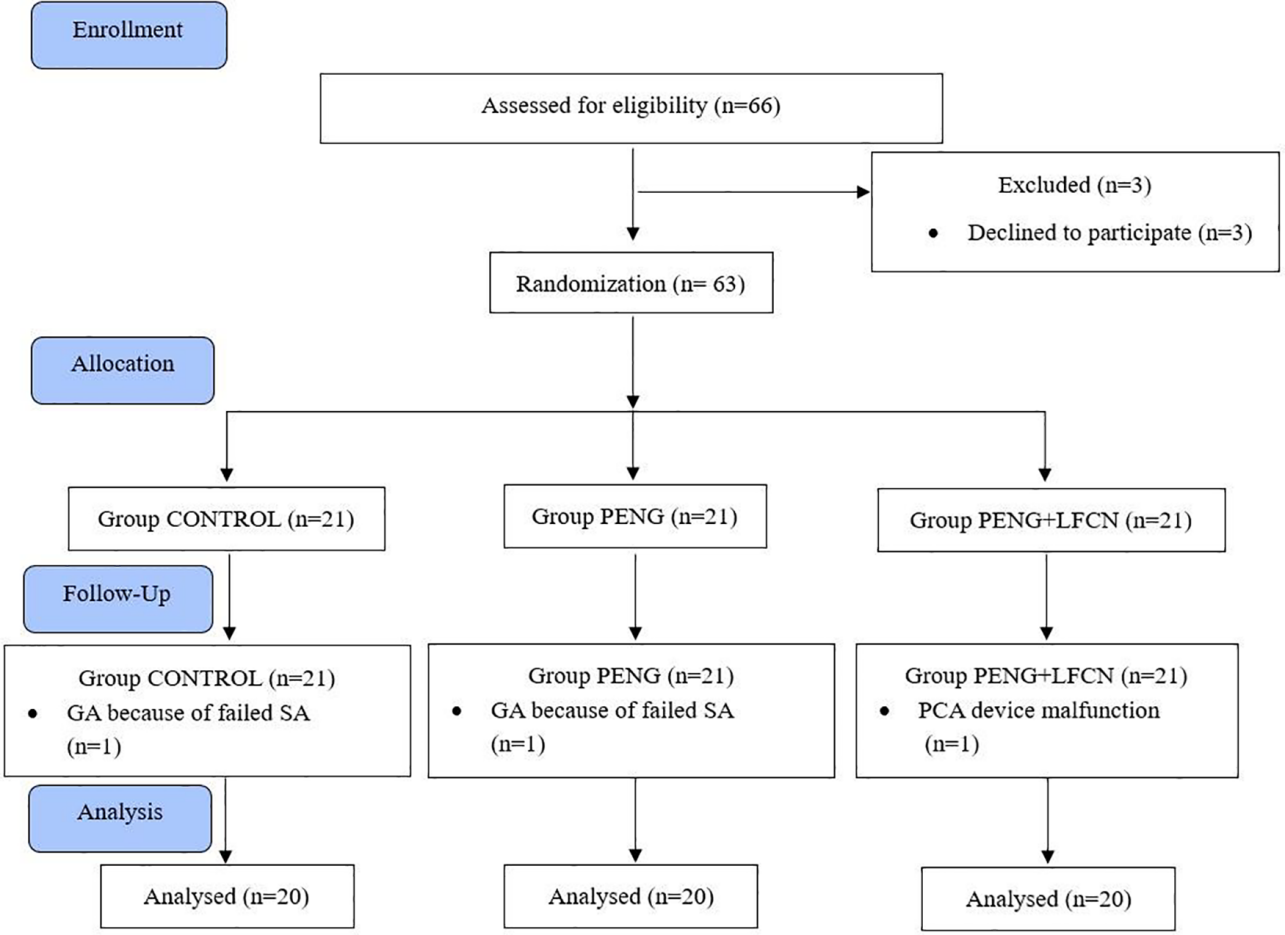
CONSORT flow diagram.

Sixty-six patients who underwent hip surgery participated in the study. Sixty patients were statistically evaluated after excluding 3 patients who refused to participate, one patient from both the CONTROL and PENG groups who received general anesthesia due to pain after spinal anesthesia, as well as one patient from the PENG + LFCN group due to technical issues with the PCA device. A total of 20 patients from each group were included in the statistical analysis (Fig. 3).

No significant differences were found in the demographic and clinical data among the groups (Table 1). Intraoperative hemodynamic parameters were also similar among the groups at all-time points.

**Table 1:** Patient demographic and clinical data.

	Group PENG (n = 20)	Group PENG + LFCN (n = 20)	Group CONTROL (n = 20)	*P*
Age	65.20 ± 1 3.95 (36–75)	63.05 ± 15.46 (18–75)	67.90 ± 9.95 (44–75)	.518
BMI	26.33 ± 5.15 (20.28–41.09)	26.06 ± 3.99 (18.59–32.20)	27.79 ± 4.92 (20.76–36.05)	.463
Sex (M/F)*	12/8	9/11	9/11	.549
Fracture side (right/left)*	13/7	11/9	10/10	.622
ASA (I/II/III)*	0/7/13	1/9/10	0/3/17	.147
Duration of anesthesia (min)	135.00 ± 16.23 (114–161)	125.90 ± 18.13 (92–156)	124.7 ± 23.41 (91–169)	.198
Duration of operation (min)	97.5 ± 13.47 (70–122)	93.65 ± 13.24 (70–121)	91.50 ± 18.25 (55–126)	.402

Data are expressed as mean ± standard deviation (SD) (min–max).

ASA = American Society of Anesthesiology, BMI = body mass index, min = minutes.

* n (number).

Postoperative total morphine consumption (IV) was significantly different among groups (PENG: 8.10 ± 6.72 mg, PENG + LFCN: 8.40 ± 4.38 mg, CONTROL: 15.30 ± 5.59 mg, *P* < .001) (Table 2).

**Table 2:** Total morphine consumption.

	Group PENG (n = 20)	Group PENG + LFCN (n = 20)	Group CONTROL (n = 20)	*P*		
Morphine consumption (mg)	8.10 ± 6.72 6 (0–32)	8.40 ± 4.38 6 (2–20)	15.30 ± 5.59 15 (8–32)	<.001		
				*PL vs P* 1	*PL vs C* <.001	*P vs C* <.001

Data are expressed as median, minimum–maximum.

C = Group CONTROL, mg = milligrams, P = Group PENG, PL = Group PENG+LFCN.

Preoperative NRS scores showed no significant differences among the groups: PENG, 1 (0–3); PENG + LFCN, 0 (0–3); CONTROL: 2 (0–4); *P* = .132. Statistically significant differences were observed among the groups at 20 minutes after the block (PENG: 0.5 [0–3], PENG + LFCN: 0 [0–2], CONTROL: 2 [0–4], *P* = .003) as well as during spinal anesthesia positioning (PENG: 2 [0–5], PENG + LFCN: 2 [0–8], CONTROL: 4.5 [0–8], *P* = .001).

A statistically significant difference was found when comparing the postoperative NRS scores at rest (NRS_İ_) and during activity (NRS_A_) among the groups (Tables 3 and 4).

**Table 3:** Comparison of postoperative resting NRS pain scores.

	Group PENG (n = 20)	Group PENG + LFCN (n = 20)	Group CONTROL (n = 20)	*P*		
*NRSR*_*0*_	0.50 (0–5)	0.00 (0–3)	2 (0–6)	.001		
				*PL vs P* .533	*PL vs C* .001	*P vs C* .048
*NRSR*_*2*_	0.50 (0–5)	1 (0–3)	2 (2–5)	<.001		
				*PL vs P* 1	*PL vs C* <.001	*P vs C* <.001
*NRSR*_*6*_	2 (0–6)	0.0 (0–3)	2 (1–5)	<.001		
				*PL vs P* .05	*PL vs C* <.001	*P vs C* .061
*NRSR*_*12*_	2 (0–2)	0.50 (0–2)	2.5 (0–4)	<.001		
				*PL vs P* .045	*PL vs C* <.001	*P vs C* .073
*NRSR*_*24*_	1 (0–4)	0.0 (0–1)	2.5 (0–3)	<.001		
				*PL vs P* .475	*PL vs C* <.001	*P vs C* <.001

Data are expressed as median and minimum–maximum.

C = Group CONTROL, NRS = numeric rating scale, NRSR = resting numerical rating scale score, NRSR_0_ = resting NRS score at 0th hour postoperatively, NRSR_2_ = resting NRS score at 2nd hour postoperatively, NRSR_6_ = resting NRS score at 6th hour postoperatively, NRSR_12_ = resting NRS score at 12th hour postoperatively, NRSR_24_ = resting NRS score at 24th hour postoperatively, P = Group PENG, PL = Group PENG+LFCN.

**Table 4:** Comparison of postoperative activity NRS pain scores.

	Group PENG (n = 20)	Group PENG + LFCN (n = 20)	Group CONTROL (n = 20)	*P*		
*NRSA*_*0*_	1 (0–6)	1 (0–4)	2 (0–7)	.026		
				*PL vs P* 1	*PL vs C* .044	*P vs C* .079
*NRSA*_*2*_	1 (0–6)	2 (0–4)	3 (2–6)	<.001		
				*PL vs P* 1	*PL vs C* .003	*P vs C* <.001
*NRSA*_*6*_	2 (0–7)	1 (0–4)	3 (1–7)	.001		
				*PL vs P* .552	*PL vs C* <.001	*P vs C* .042
*NRSA*_*12*_	2 (0–6)	1 (0–5)	3 (0–5)	.001		
				*PL vs P* .248	*PL vs C* .001	*P vs C* .182
*NRSA*_*24*_	1.50 (0–4)	1 (0–2)	3 (0–4)	<.001		
				*PL vs P* .915	*PL vs C* <.001	*P vs C* <.001

Data are expressed as median and minimum–maximum.

C = Group CONTROL, NRS = numeric rating scale, NRSA = activity numerical rating scale score, NRSA_0_ = NRS score during activity at 0th hour postoperatively, NRSA_2_ = NRS score during activity at 2nd hour postoperatively, NRSA_6_ = NRS score during activity at 6th hour postoperatively, NRSA_12_ = NRS score during activity at 12th hour postoperatively, NRSA_24_ = NRS score during activity at 24th hour postoperatively, P = Group PENG, PL = Group PENG+LFCN.

The time to the first analgesic was significantly different among the groups (PENG: 97.65 ± 47.31 minutes, PENG + LFCN: 135.85 ± 60.36 minutes, CONTROL: 49.30 ± 21.32 minutes; *P* < .001). The number of boluses requested also differed significantly (PENG: 6, PENG + LFCN: 6, CONTROL: 12; *P* < .001), as was the number of boluses given (PENG: 3, PENG + LFCN: 3, CONTROL: 7.5; *P* < .001).

No statistically significant differences in the time to first mobilization (PENG: 17 [14–26] hours, PENG + LFCN: 16.50 [12–21] hours, CONTROL: 16.50 [12–19] hours; *P* = .419) or the length of hospital stay (PENG: 5 [4–7] days, PENG + LFCN: 5 [3–8] days, CONTROL: 5.50 [3–11] days; *P* = .885) were found among the groups.

A statistically significant difference in patient satisfaction was found among the groups (*P* = .001). In the PENG group, 7 of 20 patients were very satisfied and 11 were satisfied, while in the PENG + LFCN group, 7 patients were very satisfied and 10 were satisfied. In the CONTROL group, however, no patients were very satisfied, 6 were satisfied, and 10 were undecided. No block-related complications occurred.

## 4. Discussion

In our evaluation of the effects of adding LFCN block to PENG block on postoperative opioid consumption and pain scores in patients undergoing hip fracture operation under spinal anesthesia, we found that postoperative opioid consumption was lower in the PENG and PENG + LFCN groups than in the CONTROL group, while the PENG and PENG + LFCN groups did not differ significantly. NRS pain scores were significantly lower in the PENG and PENG + LFCN groups than in the CONTROL group during spinal anesthesia positioning. In addition, the duration of the first postoperative analgesic requirement was significantly shorter in the CONTROL group than in the PENG and PENG + LFCN groups.

Three surgical approaches are used for hip fractures: anterior, posterior, and lateral.^[14]^ In our clinic, hip fractures are treated with a lateral approach by making an incision in the upper lateral thigh after closed reduction. Pascarella et al.^[13]^ demonstrated that the PENG block decreased postoperative opioid use and NRS scores in elective hip surgery. Domalgaska et al^[15]^ reported that the PENG block was effective in pain management in total hip replacement and contributed to postoperative recovery. Although the PENG block may be effective for the pain caused by the hip joint, it is not effective for the pain caused by the incision. In the lateral approach, the site of incision is innervated by the LFCN. This study aimed to investigate the effects of blocking this nerve, responsible for lateral thigh innervation, to the PENG block.

Roy et al^[16]^ found that patients who received a PENG block during hip surgery experienced pain at and below the surgical incision site postoperatively and required additional opioids. They then applied LFCN block in addition to the PENG block in 5 patients and stated that this combination provided better analgesia. Nevertheless, this result required verification by randomized controlled studies. Jadon et al^[17]^ compared PENG block with a PENG + LFCN block combination in hip fracture surgeries. They showed that the PENG block group consumed an average of 75 mg tramadol, while the PENG + LFCN combination group consumed an average of 50 mg tramadol in the first 24-hours postoperatively, which was significantly different. In our study, the total morphine consumption in the first 24-hours postoperatively was not significantly different between the PENG and PENG + LFCN groups. However, both groups consumed significantly less morphine than did the CONTROL group, suggesting that the addition of the LFCN block to the PENG block did not affect opioid consumption. We combined the blocks with paracetamol, nonsteroidal anti-inflammatory drugs, and IV morphine as part of a multimodal analgesia strategy. The reason for the lack of significant difference in the effect of addition of the LFCN block may be that the multimodal analgesia strategy effectively relieved the pain associated with skin incision.

Opioid use is almost always one of the first strategies considered for managing acute postoperative pain. However, opioids are associated with various side effects, including sedation, dizziness, nausea, vomiting, constipation, physical dependence, and respiratory depression.^[18]^ In addition, excessive opioid use has been shown to cause development of opioid tolerance as well as opioid-induced hyperalgesia.^[19]^ The goal of postoperative pain management is to minimize the need for opioids through a multimodal analgesia approach. Lin et al^[20]^ and Svraka et al^[21]^ respectively showed that the PENG block and the PENG + LFCN block significantly reduced postoperative opioid requirements in hip surgery performed under spinal anesthesia. Our study demonstrated that patients in both the PENG and PENG + LFCN groups used lower amounts of opioids than did those in the CONTROL group, indicating that PENG block were effective for analgesia in hip surgery.

Gerbershagen et al^[22]^ reported that an NRS score of ≤4 was a tolerable pain threshold in their study, which aimed to define moderate–severe postoperative pain by using NRS scores. An NRS score of 5 or higher indicates the need for rescue analgesics. Our study found no significant difference in NRS_A_ scores between the PENG and PENG + LFCN groups at any time point. A significant difference was seen between the PENG + LFCN and CONTROL groups at all-time points, as well as between the PENG and CONTROL groups at 2, 6, and 24 hours, with the values being higher in the CONTROL group. However, the average NRS_A_ scores for patients in all 3 groups were ≤3 at all-time points. The pain of all study participants was reduced to a tolerable level. A tolerable level of pain was achieved with an average of 6 mg of morphine in the PENG and PENG + LFCN groups, while the CONTROL group required 15 mg. We were thus able to present and compare the amounts of opioids consumed postoperatively objectively.

A meta-analysis examining the effects of the PENG block in hip fracture surgery under spinal anesthesia revealed that the PENG block delayed the time to first analgesic requirement.^[23]^ In our study, the time to first analgesic requirement in patients in the PENG and PENG + LFCN groups was longer than that of the CONTROL group. Although no significant difference was found between the PENG and PENG + LFCN groups, we observed that PENG + LFCN application further delayed the duration to first analgesic requirement. Additionally, analysis of the PCA data revealed that the CONTROL group requested bolus doses more frequently and in greater amounts than did the other groups.

Severe pain may occur during positioning for spinal anesthesia in patients with hip fractures. This may prevent the patient from taking up the optimum position and may decrease the chance of success of the technique. Studies have shown that block application before spinal anesthesia provides more comfortable positioning in patients with hip fractures.^[4,24]^ In our study, patients in the CONTROL group experienced moderate pain, with an average NRS score of 4.5, while those who received the PENG block had an average NRS score of 2. We consider that the PENG block may be preferred prior to neuraxial anesthesia in patients with hip fractures, in order to enhance patient comfort and to ensure that the procedure is performed under more optimal conditions.

Early mobilization after hip fracture surgery has positive effects on postoperative functional conditions, such as delirium, pain scores, length of hospital stay, pulmonary infection, and deep vein thrombosis.^[25,26]^ Therefore, techniques that relieve pain without causing motor block should be prioritized. PENG block provides effective analgesia without motor blockade by blocking the articular branches of the femoral nerve and the obturator nerve, which provide sensory innervation of the anterior capsule of the hip joint.^[27]^ In some studies, it has been shown that the use of volumes larger than 20 mL may cause the local anesthetic to spread along the intermuscular plane between the pectineus and psoas and cause undesired motor block by affecting the motor branches of the femoral nerve, and may also affect the motor branches of the obturator nerve by spreading along the lateral wall in the pelvis.^[28,29]^ In our study, a PENG block was induced with 20 mL of local anesthetic and no motor weakness developed in any of our patients. In addition, we found no difference in the time to first mobilization between our patients who received the PENG block and the CONTROL group, and all patients in the study achieved mobility within the first 24 hours.

Since the PENG block is a relatively new procedure, block-specific complications are not very clear. However, it is known that the risk of femoral nerve and vascular damage is minimal.^[30]^ It has been reported that very rare and clinically insignificant complications may occur after LFCN block. Applying the blocks under ultrasound guidance further reduces the risks.^[31]^ In our study, no complications related to the blocks developed. Our results show that blocks can be safely applied to reduce opioid consumption.

Our study has some limitations. Patients were followed-up for 24-hours postoperatively. The first limitation is that the long-term effects of the blocks were not investigated. We believe that it is important to conduct future studies with longer follow-up periods to evaluate the effects of blocks on functional recovery. Another limitation is that the duration of application and the number of attempts of performing spinal anesthesia was not evaluated. Moreover, the patients were not blinded to group allocation and the quality of recovery in the postoperative period was not measured with various scales or questionnaires.

## 5. Conclusion

We determined that the addition of a LFCN block to the PENG block in patients undergoing hip fracture surgery under spinal anesthesia did not contribute to postoperative opioid consumption and pain scores. We observed that similar pain scores were achieved with less opioids in both block groups than in the CONTROL group. We believe that preoperative PENG block plays an important role in analgesia management both during positioning for spinal anesthesia and in the postoperative period in patients undergoing hip surgery.

## Author contributions

**Conceptualization:** Dinçer Firat Şeker, Keziban Bollucuoğlu.

**Data curation:** Dinçer Firat Şeker, Bengü Gülhan Köksal İncegül, Merve Sena Baytar.

**Formal analysis:** Keziban Bollucuoğlu, Rahşan Dilek Okyay, Merve Sena Baytar.

**Investigation:** Çağdaş Baytar, Bengü Gülhan Köksal İncegül.

**Methodology:** Dinçer Firat Şeker, Keziban Bollucuoğlu.

**Project administration:** Keziban Bollucuoğlu.

**Resources:** Rahşan Dilek Okyay, Bengü Gülhan Köksal İncegül.

**Software:** Rahşan Dilek Okyay.

**Supervision:** Çağdaş Baytar.

**Visualization:** Çağdaş Baytar, Merve Sena Baytar.

**Writing – original draft:** Dinçer Firat Şeker, Keziban Bollucuoğlu.

**Writing – review & editing:** Özcan Pişkin, Hilal Ayoğlu.
